# Supplementary material for: Content-rich biological network constructed by mining PubMed abstracts
Source: BMC Bioinformatics. 2004 Oct 8;5:147. doi: 10.1186/1471-2105-5-147 (PMC528731; doi:10.1186/1471-2105-5-147)
Supplement: Additional File 2 — The original results of the above study (non-essential files are deleted to keep the file size under the limit set by BMC bioinformatics). [file 1471-2105-5-147-S2.bz2 › chilibotAdditionalFile2/dip05/531ID9671457E10848/html/HMGB2_PGR.html]

 


 **HMGB2** and **PGR** 
  
Found 2 abstracts in PubMed, retrieved 2.  
 

 What does Google say? 
 PDF only 
| .edu only 

---

- Mol Cell Biol, 1998   **High mobility group chromatin proteins 1 and 2 functionally interact with steroid hormone receptors to enhance their DNA binding in vitro and transcriptional activity in mammalian cells.** .
  We previously reported that the chromatin high mobility group protein 1 HMG 1 enhances the sequence specific DNA binding activity of progesterone receptor **[PGR]** PR in vitro, thus providing the first evidence that HMG 1 may have a coregulatory role in steroid receptor mediated gene transcription.
  Here we show that HMG 1 and the highly related HMG 2 **[HMGB2]** stimulate DNA binding by other steroid receptors, including estrogen, androgen, and glucocorticoid receptors, but have no effect on DNA binding by several nonsteroid nuclear receptors, including retinoid acid receptor RAR , retinoic X receptor RXR , and vitamin D receptor VDR .
  As highly purified recombinant full length proteins, all steroid receptors tested exhibited weak binding affinity for their optimal palindromic hormone response elements HREs , and the addition of purified HMG 1 or 2 substantially increased their affinity for HREs.
  Purified RAR, RXR, and VDR also exhibited little to no detectable binding to their cognate direct repeat HREs but, in contrast to results with steroid receptors, the addition of HMG 1 or HMG 2 **[HMGB2]** had no stimulatory effect.
  Instead, the addition of purified RXR enhanced RAR and VDR DNA binding through a heterodimerization mechanism and HMG 1 or HMG 2 **[HMGB2]** had no further effect on DNA binding by RXR RAR or RXR VDR heterodimers.
  HMG 1 and HMG 2 **[HMGB2]** HMG 1 2 themselves do not bind to progesterone response elements, but in the presence of PR they were detected as part of an HMG PR DNA ternary complex.
  HMG 1 2 can also interact transiently in vitro with PR in the absence of DNA.
  no direct protein interaction was detected with VDR.
  These results, taken together with the fact that PR can bend its target DNA.
  HMG 1 2 are non sequence specific DNA binding proteins that recognize DNA structure, suggest that HMG 1 2 are recruited to the PR DNA complex by the combined effect of transient protein interaction and DNA bending.
  In transient transfection assays, coexpression of HMG 1 or HMG 2 **[HMGB2]** increased PR mediated transcription in mammalian cells by as much as 7 to 10 fold without altering the basal promoter activity of target reporter genes.
  This increase in PR mediated gene activation by coexpression of HMG 1 2 was observed in different cell types and with different target promoters, suggesting a generality to the functional interaction between HMG 1 2 and PR in vivo.
  Cotransfection of HMG 1 also increased reporter gene activation mediated by other steroid receptors, including glucocorticoid and androgen receptors, but it had a minimal influence on VDR dependent transcription in vivo.
  These results support the conclusion that HMG 1 2 are coregulatory proteins that increase the DNA binding and transcriptional activity of the steroid hormone class of receptors but that do not functionally interact with certain nonsteroid classes of nuclear receptors.

  - J Biol Chem, 1995   **Increased DNA bending activity and higher affinity DNA binding of high mobility group protein HMG 1 prepared without acids.** .
    Recently, DNA ring closure assays showed that high mobility group protein HMG 1 and its close homolog HMG 2 **[HMGB2]** mediate sequence independent DNA flexion.
    This DNA bending activity appears to be central to at least some of the recently elucidated functions of HMG 1 2, such as the enhancement of progesterone receptor **[PGR]** DNA binding.
    Here we show that standard purification procedures utilizing perchloric and trichloroacetic acid can produce HMG 1 significantly deficient in its abilities to bind and bend double stranded DNA, while acid independent methods purify HMG 1 that is superior in these respects.
    Significant losses of DNA ring closure activity were seen upon limited 2 5 h exposures of nonacid purified HMG 1 2 to perchloric acid and or trichloroacetic acid.
    Measurements of the apparent DNA dissociation binding constant Kd app of acid extracted preparations of HMG 1 gave a wide range of values, and only those preparations demonstrating little DNA ring closure activity had Kd values near the previously published value approximately 10 6 M .
    The highest ring closure activities and lowest Kd app <.
    3 x 10 9 M were obtained for HMG 1 purified without acids.
    These combined results support the use of alternative, non acid purification procedures for preserving the DNA bending activity of HMG 1 2 and suggest that past procedures utilizing acids have led to an underestimation of the affinity of HMG 1 for DNA.
